# Early changes in cell‐free DNA levels in newly transplanted heart transplant patients

**DOI:** 10.1111/petr.13622

**Published:** 2019-12-11

**Authors:** Steven D. Zangwill, Steven J. Kindel, William S. Ragalie, Paula E. North, Alyssa Pollow, Mats Hidestrand, Aoy Tomita‐Mitchell, Karl D. Stamm, Michael E. Mitchell

**Affiliations:** ^1^ Division of Cardiology Phoenix Children's Hospital Phoenix AZ USA; ^2^ Division of Pediatric Cardiology Medical College of Wisconsin Herma Heart Institute, Children's Wisconsin Milwaukee WI USA; ^3^ Division of Cardiothoracic Surgery David Geffen School of Medicine at UCLA Los Angeles CA USA; ^4^ Department of Pathology Medical College of Wisconsin Children's Hospital of Wisconsin Wauwatosa WI USA; ^5^ Division of Pediatric Cardiothoracic Surgery, Medical College of Wisconsin Herma Heart Institute, Children's Wisconsin Milwaukee WI USA; ^6^ Tai Diagnostics Inc. Wauwatosa WI USA

**Keywords:** biomarkers, cell-free DNA, early postoperative period, pediatric heart transplant

## Abstract

Heart transplantation is a well‐established therapy for end‐stage heart failure in children and young adults. The highest risk of graft loss occurs in the first 60 days post‐transplant. Donor fraction of cell‐free DNA is a highly sensitive marker of graft injury. Changes in cell‐free DNA levels have not previously been studied in depth in patients early after heart transplant. A prospective study was conducted among heart transplant recipients at a single pediatric heart center. Blood samples were collected from children and young adult transplant patients at three time points within 10 days of transplantation. DF and total cell‐free DNA levels were measured using a targeted method (myTAI_HEART_). In 17 patients with serial post‐transplant samples, DF peaks in the first 2 days after transplant (3.5%, [1.9‐10]%) and then declines toward baseline (0.27%, [0.19‐0.52]%) by 6‐9 days. There were 4 deaths in the first year among the 10 patients with complete sample sets, and 3 out of 4 who died had a late rise or blunted decline in donor fraction. Patients who died trended toward an elevated total cell‐free DNA at 1 week (41.5, [34‐65] vs 13.6, [6.2‐22] *P* = .07). Donor fraction peaks early after heart transplant and then declines toward baseline. Patients without sustained decline in donor fraction and/or elevated total cell‐free DNA at 1 week may have worse outcomes.

**Abbreviations**
cfDNA: cell‐free DNA
CHW: Children's Hospital of Wisconsin
DF: donor fraction
DNA: deoxyribonucleic acid
ECMO: extracorporeal membrane oxygenation
HTx: heart transplantation
MCS: mechanical circulatory support
PCR: polymerase chain reaction
QC: quality control
TcfDNA: total cell‐free DNA

## 1 INTRODUCTION

DF of cfDNA has been proposed as a stable marker for cellular injury caused by rejection in several organs including the heart.^1^, ^2^ An increased DF during episodes of rejection has been reported in both adult and pediatric heart transplant recipients.^3^, ^4^ Limited information exists regarding serial changes in DF in the immediate post‐transplant phase. De Vlaminck et al^5^ reports that DF declines to baseline at 1‐week post‐heart transplant. Similarly, in a study of DF following liver transplant, baseline levels were reached somewhere between 7 and 10 days post‐transplant.^6^ Just as DF has value as a specific marker for graft injury, the total amount of circulating cfDNA also may have diagnostic and prognostic value. TcfDNA is an emerging marker of inflammation and cell turnover with diverse applications in clinical practice.^7^, ^8^, ^9^ Herein, we report high‐resolution measurements of DF and TcfDNA at three time points during the first week post‐transplant using a rapid and economical targeted assay suitable for clinical surveillance.^10^

## 2 PATIENTS AND METHODS

A prospective study to assess the utility of DF cfDNA as a marker for rejection was conducted among heart transplant recipients at a single pediatric heart center. A substudy to characterize early changes in DF and TcfDNA after heart transplant was devised. All patients listed for cardiac transplant at the Herma Heart Center at the CHW were invited to participate in this substudy. Samples were excluded if they (a) were from multi‐organ transplant recipients, (b) failed genotyping QC, (c) had incompletely documented collection times, (d) were obtained from patients on ECMO, or (e) the transplant recipient had fewer than two DF results in the first 9 days post‐transplant. Informed consent was obtained for all subjects through a protocol approved by the CHW Institutional Review Board (CHW 10/83, GC 1111).

### 2.1 Blood sample collection

Three to ten milliliter of anti‐coagulated blood was collected to assess circulating levels of cfDNA. Each sample was collected in 10‐mL cfDNA Blood Collection Tubes (BCT) tubes (Streck). Samples were immediately coded, de‐identified, and delivered to the laboratory for processing. Blood samples were obtained from subjects at three targeted time points following transplantation, characterized as day 1: within 0‐2 days; day 4: within 3‐6 days; and day 8: within 6‐9 days of transplant.

### 2.2 Plasma processing and DNA extraction

Separation of plasma from whole blood by centrifugation was carried out as previously described.^11^ Plasma was stored at −80°C until DNA extraction. CfDNA extractions were performed using ReliaPrep^TM^ HT Circulating Nucleic Acid Kit, Custom (Promega). Recipient genomic DNA was extracted by using ReliaPrep^TM^ Large Volume gDNA Isolation System (Promega) or Gentra Puregene Blood Kit (Qiagen).

### 2.3 Total cfDNA analysis

Total cfDNA (TcfDNA) content from plasma was evaluated by quantitative real‐time PCR as previously described.^11^ PCR analysis was carried out on an Applied Biosystems QuantStudio 7 Flex Real‐Time PCR System (Thermo Fisher Scientific). A dilution series of human genomic DNA was used to create a standard curve for quantification.

### 2.4 Quantitative genotyping

The myTAI‐HEART™ assay was used to calculate DF as a percentage of TcfDNA (TAI Diagnostics).^10^ The assay quantitatively genotypes a panel of high‐frequency single‐nucleotide polymorphisms selected for their ability to reliably discriminate between alleles and was performed without a donor sample.^10^

### 2.5 Clinical data collection

Clinical, laboratory, cardiac catheterization, and echocardiographic data were recorded at the time of collection, and data were managed using Research Electronic Data Capture (REDCap) tools hosted at CHW.^12^ Data reviewed included demographics and key operative characteristics including ischemic time and bypass time. Outcomes measured include recipient and graft outcomes, death, treatment for rejection, biopsy results, treatment for infection, MCS, length of hospital stay post‐transplant, follow‐up survival time, and any known cardiac arrest episodes. Additional verified information includes primary cause of death for subjects who died, ischemic time in minutes, and patient age.

### 2.6 Statistical analysis

Data processing was performed in RStudio, statistical analyses include linear modeling, Wilcoxon's rank‐sum test, and the Student's *t* test for difference in means. Data are reported as median and interquartile range.

## 3 RESULTS

**Figure 1:**
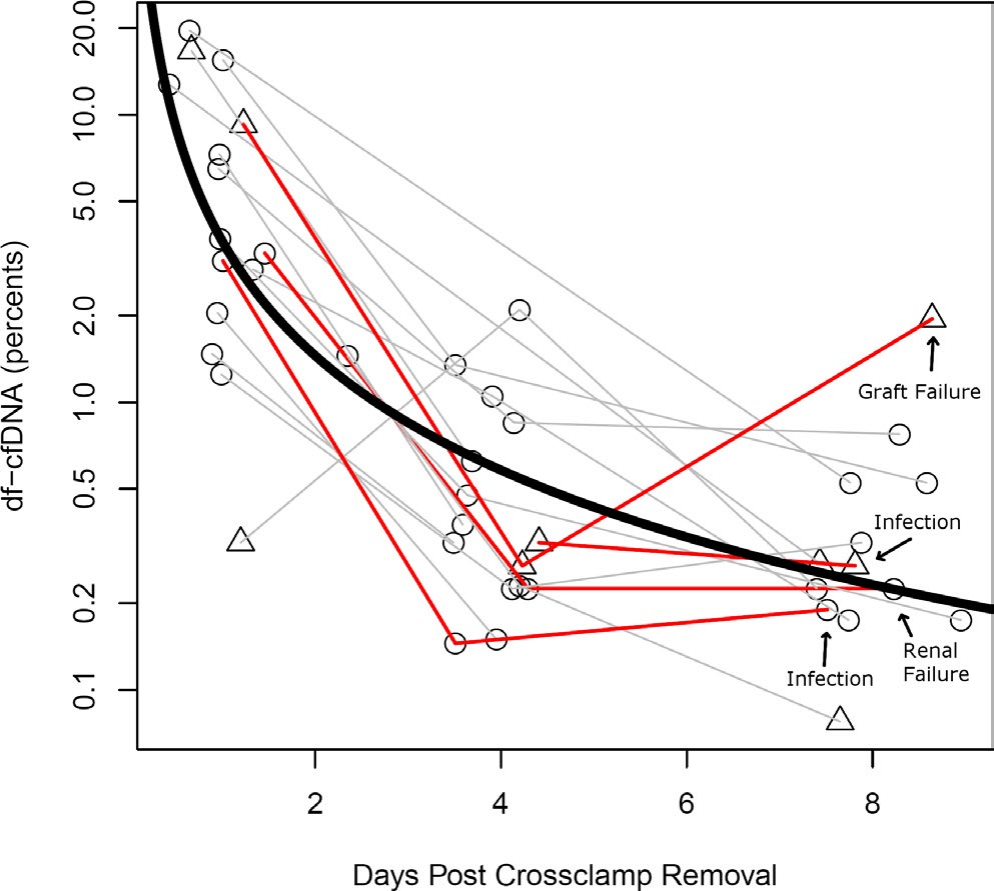
DF cfDNA measured at time points immediately after transplant. Time points are calculated to the minute when available and correspond to targeted post‐transplant days 1, 4, and 8. Circles indicate samples drawn without indication of infection, triangles indicate active treatment for infection. Gray lines (n = 13) indicate trajectories of patients surviving the first year, and red lines indicate those who died (n = 4). Thick black curve is a linear trend line averaging all data points (normal decline)

Seventeen patients were identified with at least two samples within the targeted time frame 0‐9 days post‐transplant. One sample was excluded for failure to pass genotyping QC. Patient characteristics are shown in Table 1. The median age was 4.1 with a range of 0.2‐23.8 years. The majority were status 1A and male and had congenital heart disease at the time of transplant. None were on dialysis and four required MCS going into transplant. Figure 1 shows how DF for each patient evolves after transplant. In general, each day post‐transplant was associated with significant decrease in DF with a linear fit: *y* = −1.3 × −1.4, (where y is log10 of DF, and *x* is log10 days post‐transplant, both coefficients significant *P* < .01). Median (IQR) DF at day 1 was 3.5% [1.9‐10%], day 4 was 0.325% [0.23‐0.74%], and day 8 was 0.27% [0.19‐0.52%]. Using the median DF of 0.25% previously determined using our analytical method for patients with negative endomyocardial biopsies,^10^ we found that the measured DF approaches baseline by the end of the first week post‐transplant. We identified patients undergoing active treatment for infection (Figure 1, circles vs triangles). Treatment for infection was defined as documented anti‐infective therapy other than standard post‐transplant prophylaxis regimens. There was no difference in DF based on treatment for infection.

**Table 1:** Patient's characteristics

	Patient Cohort (n = 17)
Age (y)	4.1 (0.3‐12.5)
Sex	
Male	11 (65%)
Female	6 (35%)
Diagnosis	
Congenital heart disease	10 (59%)
Cardiomyopathy	7 (41%)
Ischemic time (min)	206 (171‐226)
Bypass time (min)	175 (159‐241)
Status at heart transplant	
1A	12 (71%)
1B	5 (29%)
MCS at heart transplant	4 (24%)
Berlin Heart	2 (12%)
HeartWare	1 (6%)
HeartMate II	1 (6%)
Dialysis at heart transplant	None
MCS post‐heart transplant	2 (12%)—ECMO

Note

Data expressed as median (interquartile range).

Recipient and graft outcomes were analyzed for patients with three DF results. Ten of these patients had samples available for three time points and were selected for further review of patient demographics and key operative characteristics including ischemic time and bypass time. Six out of the ten patients with three time points had a decline in their DF between days 4 and 8, all survived the first 20 months. The four remaining patients had non‐declining DF, and only one of those survived the first year. There was no significant relationship between first DF result and ischemic times or bypass times.

Thirteen patients had measurements before and after day 6. Four of thirteen did not survive the first year and had significantly reduced or no decline in DF (+0.1%/d) vs the nine of thirteen who survived the first year (−0.5%/d, *P* = .03). Clinical outcomes for all patients were followed and, notably, the one patient whose DF remained high (>1%) 8 days post‐transplant was the single individual in the study who died of graft failure in the first year post‐transplant (day 55).

**Figure 2:**
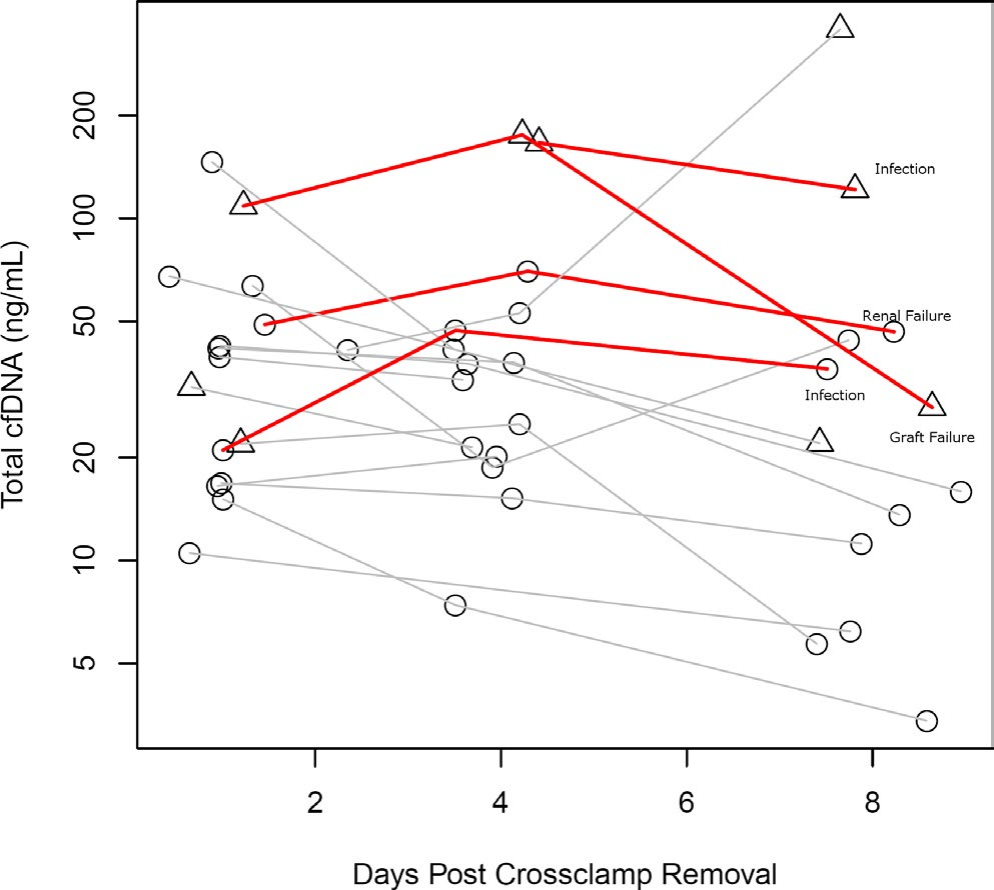
Circles indicate samples drawn without indication of infection, and triangles indicate active treatment for infection. Black lines indicate trajectories of patients surviving the first year, and red solid lines indicate those who died

Changes in TcfDNA in the early post‐transplant period are shown in Figure 2. Again, we differentiated patients based on whether they were undergoing treatment for infection. TcfDNA trended higher in patients who died compared with those who survived (41.5, [34‐65] vs 13.6, [6.2‐22] *P* = .07). Four of six patients with TcfDNA >25 ng/mL at 1‐week post‐transplant did not survive the first year (Figure 2). Three of four patients who died had both an increase in DF between days 4 and 8 and a persistently elevated level of TcfDNA, and the fourth non‐survivor had a trivial decline in DF from days 4 to 8. Of 13 patients with TcfDNA levels around day 8 post‐transplant, 4 were undergoing treatment for infection and had elevated TcfDNA levels compared with patients not undergoing infection treatment (75 [26‐180 ng/mL] vs 14 [6.2‐36 ng/mL], *P* = .038).

## 4 DISCUSSION

DF of circulating cfDNA is a highly sensitive marker of graft injury following HTx. Monitoring of DF in HTx recipients has been shown in a growing number of studies to be a promising screening tool for rejection.^3^, ^4^, ^5^, ^10^ Given the fact that DF can be performed more frequently than biopsy, its availability could empower early intervention and improve graft viability. Our data align with previous reports that DF approaches baseline around 1 week following the heart transplant procedure.^5^ This early and predictable decline in DF renders it useable as a screening tool for rejection in the early post‐transplant period; this is important as the first few months represent a period of increased risk. Further, alternative non‐invasive tools such as gene expression profiling are not approved for use during this highly vulnerable time frame.

While others have shown a similar decline in DF over the first week post‐HTx, the addition of serial testing during the first week and the corresponding analysis of TcfDNA levels provides additional value to the early assay results. This study documents for the first time the pattern of decline of DF and total cfDNA level in the first week (8 days) post‐heart transplant surgery. In this series, there were 4 patients who died within the first year, 3 during their transplant hospitalization; all had some combination of a rise (or blunted decline) in DF from days 4 to 8 and/or a persistently elevated TcfDNA at 1‐week post‐transplant. TcfDNA can be elevated under many circumstances particularly those associated with inflammation and conditions of high cell turnover. Emerging literature across many disciplines have shown the prognostic significance of elevated TcfDNA levels in diverse clinical settings.^7^, ^8^, ^9^ The early period post‐HTx is a complex environment when trying to characterize the expected patterns of DF and TcfDNA levels. The donor organ may be injured in the primary event that led to donor candidacy; this is followed by obligate cold ischemia and direct surgical trauma in the setting of cardiopulmonary bypass (a highly pro‐inflammatory event). The clinical course then over the first week may be highly variable and subject to a myriad of factors potentially affecting release and clearance of circulating cfDNA. TCF levels were on average higher in the first 8 days for patients who died within the first year post‐transplant, although the small sample number in this study precludes demonstration of statistical significance of this association. This may be explained by the fact that TcfDNA is a general indicator of non‐cardiac as well as cardiac cellular injury and would be expected to correlate with illness preceding death. In this study, we found that the pattern of decline in DF during the first post‐transplant week along with the level of TcfDNA may be prognostically important. Marked elevation of TcfDNA at 1 week following HTx and a rise in DF from days 4 to 8 are associated with poor outcomes. Larger studies are needed to further understand the relationship and prognostic significance of TcfDNA and DF early post‐heart transplant.

## 5 LIMITATIONS

This study should be considered in the context of a few important limitations. This analysis was part of a pilot study focused on the relationship between DF cfDNA and rejection in cardiac transplant recipients; this substudy evaluating early changes in cfDNA following heart transplant included <20 patients. Further, all of the subjects were pediatric or young adult recipients with corresponding young donors. The study cohort as a whole had an uncharacteristically high rate of death. While we could not identify a source of selection bias, it will be important to validate these trends in a larger more representative series of heart transplant recipients. Finally, the draw times for serial sampling was not consistent but rather fell within ranges as described in the manuscript.

## CONFLICT OF INTEREST

A.T‐M. and MM own stock and are co‐founders of TAI Diagnostics. KS owns stock and is an employee of TAI Diagnostics. SZ and MH own stock and are consultants to TAI Diagnostics.

## References

[1] Hubacek JA , Vymetalova Y , Bohuslavova R , Kocik M , Malek I . Detection of donor DNA after heart transplantation: how far could it be affected by blood transfusion and donor chimerism? Transplant Proc. 2007;39(5):1593‐1595. 17580196 10.1016/j.transproceed.2007.01.093

[2] Lo YM , Tein MS , Pang CC , Yeung CK , Tong KL , Hjelm NM . Presence of donor‐specific DNA in plasma of kidney and liver‐transplant recipients. Lancet. 1998;351:1329‐1330. 9643800 10.1016/s0140-6736(05)79055-3

[3] Hidestrand M , Tomita‐Mitchell A , Hidestrand PM , et al. Highly sensitive noninvasive cardiac transplant rejection monitoring using targeted quantification of donor‐specific cell‐free deoxyribonucleic acid. J Am Coll Cardiol. 2014;63:1224‐1226. 24140666 10.1016/j.jacc.2013.09.029 PMC4988656

[4] Snyder TM , Khush KK , Valantine HA , Quake SR . Universal noninvasive detection of solid organ transplant rejection. Proc Natl Acad Sci USA. 2011;108:6229‐6234. 21444804 10.1073/pnas.1013924108 PMC3076856

[5] De Vlaminck I , Valantine HA , Snyder TM , et al. Circulating cell‐free DNA enables noninvasive diagnosis of heart transplant rejection. Sci Transl Med. 2014;6:241ra77. 10.1126/scitranslmed.3007803 PMC4326260 24944192

[6] Schutz E , Fischer A , Beck J , et al. Graft‐derived cell free DNA, a non‐invasive early rejection and graft damage marker in liver transplantation: a prospective observational, multicenter cohort study. PLoS Med. 2017;25:14(4). 10.1371/journal.pmed.1002286 PMC5404754 28441386

[7] Valpione S , Gremel G , Mundra P , et al. Plasma total cell‐free DNA (cfDNA) is a surrogate biomarker for tumour burden and a prognostic biomarker for survival in metastatic melanoma patients. Eur Journal of Cancer. 2018;88:1‐9. 10.1016/j.ejca.2017.10.029 PMC5769519 29175734

[8] Shoham Y , Krieger Y , Perry ZH ,, et al. Admission cell free DNA as a prognostic factor in burns: quantification by use of a direct rapid fluorometric technique. Biomed Research Intl. 2014;2014:1‐5. 10.1155/2014/306580 PMC4090497 25045663

[9] Gögenur M , Burcharth J , Gögenur I . The role of total cell‐free DNA in predicting outcomes among trauma patients in the intensive care unit: a systematic review. Crit Care. 2017;21(1):14. 28118843 10.1186/s13054-016-1578-9 PMC5260039

[10] Ragalie WS , Stamm K , Mahnke D , et al. Noninvasive assay for donor fraction of cell‐free DNA in pediatric heart transplant recipients. J Am Coll Cardiol. 2018;71:2982‐2983. 29929623 10.1016/j.jacc.2018.04.026

[11] Hidestrand M , Stokowski R , Song K , et al. Influence of temperature during transportation on cell‐free DNA analysis. Fetal Diagn Ther. 2012;31:122‐128. 22261730 10.1159/000335020

[12] Harris PA , Taylor R , Thielke R , Payne J , Gonzalez N , Conde JG . Research electronic data capture (REDCap)–a metadata‐driven methodology and workflow process for providing translational research informatics support. J Biomed Inform. 2009;42:377‐381. 18929686 10.1016/j.jbi.2008.08.010 PMC2700030

